# Participation of NADPH Oxidase-Related Reactive Oxygen Species in Leptin-Promoted Pulmonary Inflammation: Regulation of cPLA2α and COX-2 Expression

**DOI:** 10.3390/ijms20051078

**Published:** 2019-03-02

**Authors:** Pei-Sung Hsu, Chia-Mo Lin, Jia-Feng Chang, Chi-Sheng Wu, Kee-Chin Sia, I-Ta Lee, Kuo-Yang Huang, Wei-Ning Lin

**Affiliations:** 1 Division of Chest Medicine, Shin Kong Wu Ho-Su Memorial Hospital, Taipei 111, Taiwan; pack14tw@gmail.com (P.-S.H.); aminus64@gmail.com (C.-M.L.); 2 Department of Chemistry, Fu-Jen Catholic University, New Taipei City 242, Taiwan; 3 Department of Internal Medicine, En-Chu-Kong Hospital, New Taipei City 237, Taiwan; cjf6699@gmail.com; 4 Department of Medical Research, Taichung Veterans General Hospital, Taichung 407, Taiwan; itl700128@gmail.com; 5 Department of Nursing, College of Nursing, Hungkuang University, Taichung 433, Taiwan; 6 Graduate Institute of Pathology and Parasitology, National Defense Medical Center, Taipei 114, Taiwan; cguhgy6934@gmail.com; 7 Graduate Institute of Biomedical and Pharmaceutical Science, Fu Jen Catholic University, New Taipei City 242, Taiwan; linweining@ms40.url.com.tw (C.-S.W.); abangsia3648@gmail.com (K.-C.S.)

**Keywords:** leptin, cPLA2α, COX-2, ROS, c-Jun, inflammation

## Abstract

Obesity is a worldwide epidemic problem and correlates to varieties of acute or chronic lung diseases such as acute respiratory distress syndrome, chronic obstructive pulmonary disease, and pulmonary fibrosis. An increase of leptin, a kind of adipokine, in lean mice plasma has been found to impair immune responses and facilitate the infection of *Klebsiella pneumoniae*, resulting in increased pneumonia severity. Also, a higher leptin level is found in exhaled breath condensates of obese or asthmatic subjects, compared to healthy ones, suggesting that leptin is involved in the occurrence or exacerbation of lung injury. In previous studies, we showed that leptin stimulated cytosolic phospholipase A2-α (cPLA2α) gene expression in lung alveolar type II cells via mitogen-activated protein kinase (MAPK) and nuclear factor-kappa B (NF-κB)-activated coactivator p300. Herein, we show that the in vivo application of leptin in the respiratory system upregulated the expression of inflammatory proteins cPLA2α and cyclooxygenase-2 (COX-2) together with leukocyte infiltration. Treatment with an ROS scavenger (*N*-acetylcysteine, NAC), an NADPH oxidase inhibitor (apocynin), or an activating protein (AP)-1 inhibitor (tanshinone IIA) attenuated leptin-mediated cPLA2α/COX-2 expression and leukocyte recruitment in the lung. Leptin increased intracellular oxidative stress in a leptin receptor (OB-R) and NADPH oxidase-dependent manner, leading to the phosphorylation of the AP-1 subunit c-Jun. In summation, leptin increased lung cPLA2α/COX-2 expression and leukocyte recruitment via the NADPH oxidase/ROS/AP-1 pathway. Understanding the inflammatory effects of leptin on the pulmonary system provides opportunities to develop strategies against lung injury related to metabolic syndrome or obesity.

## 1. Introduction

An imbalance of energy intake and consumption, results in the occurrence of obesity. Obesity has been found to correlate to varieties of lung diseases such as asthma, acute respiratory distress syndrome (ARDS), chronic obstructive pulmonary disease (COPD), pulmonary fibrosis, and sleep apnea [1]. Patients with both ARDS and obesity have longer durations of mechanical ventilation and intensive care unit stays [1], and compared with normal weight patients, the morbidity of ARDS/acute lung injury (ALI) is higher in obese patients [2]. Increased ratio of wet/dry lung weight, increased expression of inducible nitric oxide synthase (iNOS), and a reduction in blood oxygen saturation were found in 30% fat diet-fed female mice, but not in standard low-fat diet-fed control groups after intestinal ischemia and reperfusion [3]. This suggested that the lung protection effects of estrogen in female mice were attenuated in obese groups, and obesity predisposes female mice to pulmonary edema, iNOS expression, and exacerbation of gas exchange in the lung [3]. In comparison with the serum effects from lean mice, treatment of lung endothelial cells with obese serum promoted adhesion protein expression and reduced junctional protein expression, resulting in an increased susceptibility to lipopolysaccharide-regulated lung injury [4]. In lipopolysaccharide (LPS)-challenged ALI mice, obesity increased lung recruitment of neutrophils via up-regulated lymphocyte function-associated antigen 1 and intracellular adhesion molecule-1 (ICAM-1) expression [5]. Similarly, obesity in moderate to severe COPD patients correlates to a worse COPD-related course, and increased morbidity [6]. Although several studies have reported an association between obesity and pulmonary diseases, the molecular mechanisms have been less discussed.

Inflammation is companied with various pulmonary diseases including ARDS, ALI, COPD, and sleep apnea [7]. Cytosolic phospholipase A2-α (cPLA2α) and cyclooxygenase-2 (COX-2) are pro-inflammatory proteins and participate in the generation of eicosanoids. Arachidonic acid is produced by cPLA2α via the hydrolysis of membrane glycerophospholipids at the sn-2 position. COX-2 further converts arachidonic acid into eicosanoids, including prostaglandins (PGs) [7]. It was found that the expression of cPLA2α and COX-2 is related to the occurrence or amplification of inflammation [8]. Inhibition of cPLA2α by methyl arachidonyl fluorophosphonate attenuates benzo[a]pyrene in PM_2.5_-induced pulmonary injury and IL-8 release in alveolar type II A549 cells [9]. COX-2 has been proved to play roles in cockroach-extract-regulated lung inflammation and asthma [10]. An inhibitor of COX-2, celecoxib, attenuated inflammatory gene expressions and chemotaxis in adipose tissue, suggests the participation of COX-2 in obesity-induced inflammation of the adipose tissue [11]. In addition, cPLA2α and COX-2 are involved in the tissue recruitment of leukocytes [12,13], suggesting roles for both proteins in inflammation. Whether obesity contributes to lung inflammation via regulating cPLA2α/COX-2 expression and the related mechanism has not been fully addressed.

Leptin is a 16 kDa adipokine encoded in human chromosome 7q31.3 and is secreted mainly by enlarged adipose tissue [14]. Several studies have reported that leptin provides a link between obesity and lung diseases. An increase of leptin in lean mice plasma impairs immune responses and facilitates the infection of Klebsiella pneumoniae, resulting in increased pneumonia severity [15]. It has been reported that there are higher leptin levels in the exhaled breath condensates of obese or asthmatic subjects compared to healthy ones [16]. In addition, leptin directly binds to the leptin receptor (OB-R) and activates nuclear factor-kappa B (NF-κB), resulting in the increased expression of ICAM-1, C-C motif chemokine ligand 11 (CCL11), vascular endothelial growth factor (VEGF), granulocyte colony stimulating factor (G-CSF), and IL-6 in human airway epithelial cells [17]. Upregulation of migration, inhibition of apoptosis, and increased proliferation are also found in leptin-stimulated airway epithelial cells [17]. Leptin also facilitates the production and secretion of mucin 5AC (MUC5AC) protein via IL-13-activated JAK2–STAT3 in the human bronchial epithelial cell line-16 (HBE16) cells [18]. In our previous work, leptin promoted the expression of the cPLA2α gene in lung alveolar type II cells via MAPK and NF-κB-activated coactivator p300 [19]. Whether leptin participates in pulmonary inflammation in vivo and the related molecular mechanisms are still not fully studied.

Reactive oxygen species (ROS) play roles in various physiological and pathological conditions promoting bacteria scavenging and functioning as intracellular signaling molecules [20,21]. Activation of NADPH oxidase is one source of intracellular ROS production [22]. It was found that the inhibition of NADPH oxidase by apocynin attenuates severe trauma–related lung injury in obese rats [23]. NADPH oxidase-mediated ROS also contribute to cytokine-mediated inflammatory protein expression, such as of adhesion molecules, cPLA2α, and COX-2 [7,24]. Moreover, dimerization of c-Jun with c-Fos functions as a transcription factor activator protein-1 (AP-1) promoting gene activation. It was found that activation of AP-1 contributed to cPLA2α and COX-2 expression in human tracheal smooth muscle cells and lung epithelial cells [25,26]. Activation of AP-1 has also been found in leptin-mediated insulin-like growth factor (IGF)-1 transcription in human breast cancer cells [27]. Whether ROS-regulated AP-1 activation is involved in leptin-regulated cPLA2α and COX-2 expression in vivo has not been fully addressed. In this study, the effects of leptin on regulating cPLA2α and COX-2 expression, and the related molecular mechanisms, were tested in vivo in Institute of Cancer Research (ICR) mice.

## 2. Results

### 2.1. Leptin Promotes cPLA2α and COX-2 Expression in Lung Tissue

**Figure 1:**
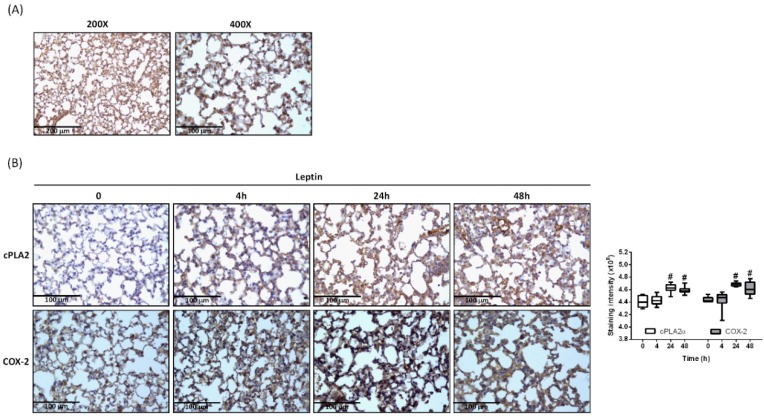
Leptin induced expression of cytosolic phospholipase A2-α (cPLA2) and cyclooxygenase-2 (COX-2) in the lung. (**A**) Immunohistochemistry (IHC) staining of leptin receptor (OB-R) in the lung section of Institute of Cancer Research (ICR) mice. (**B**) ICR mice were intra-tracheally (i.t.) injected with 2 mg/Kg of leptin for 0, 4, 24, or 48 h. At the end of incubation, mice were sacrificed, and lung tissue was extracted and paraffinized. Expression of cPLA2 (top) and COX-2 (bottom) in lung tissues was detected by IHC staining. The staining intensity of cPLA2 or COX-2 is shown as box plots summarizing the median, 25th and 75th percentiles, whiskers, and outliers. ^#^ *p* < 0.05 as compared with the group of 0 min.

Obesity is accompanied by increased leptin concentration in plasma [28]. In addition, high numbers of ^125^I-monoiodoleptin analog binding sites appear at the pulmonary parenchyma and bronchiolar epithelial level of the lung [29]. In our previous work, expression of leptin receptor (OB-R) mRNA was also demonstrated on lung type II epithelial cells [19]. Here, we show that OB-R was expressed in lung tissue by immunohistochemistry (IHC) staining (Figure 1A). Mice were intra-tracheally injected with leptin for 0, 4, 24, or 48 h. At the end of incubation, mice were sacrificed and lung tissues were paraffin embedded. IHC staining was performed to evaluate the expression of cPLA2α and COX-2 protein. Stimulation with leptin increased protein expression of cPLA2α and COX-2 in lung tissues (Figure 1B).

### 2.2. Participation of ROS in Leptin-Promoted cPLA2α and COX-2 Expression

**Figure 2:**
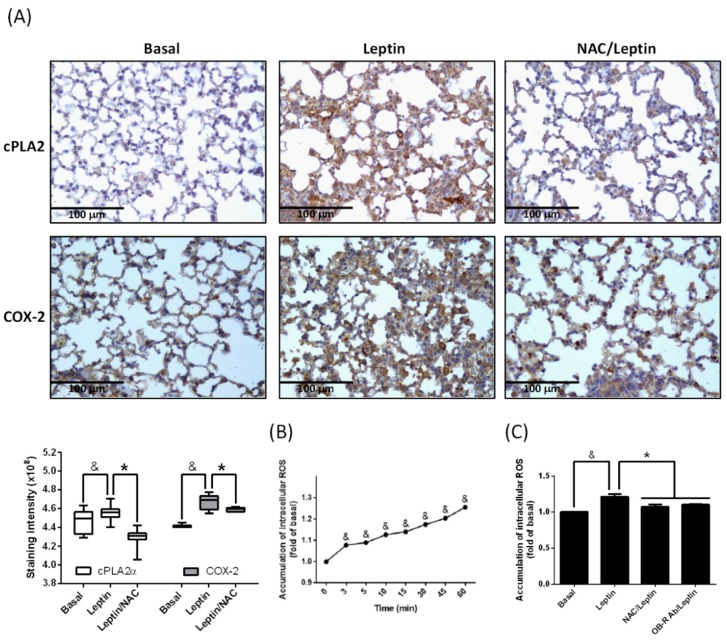
Participation of reactive oxygen species (ROS) in leptin-upregulated expression of cPLA2 and COX-2. (**A**) ICR mice were intraperitoneally (i.p.) injected with 2 mg/Kg of *N*-acetylcysteine (NAC) for 1 h then i.t. injected with 2 mg/Kg of leptin for 48 h. Expression of cPLA2 (top) and COX-2 (bottom) were shown by IHC staining. The staining intensities of cPLA2 and COX-2 are shown as box plots summarizing the median, 25th and 75th percentiles, whiskers, and outliers. (**B**) A549 cells were treated with 1 μg/mL of leptin for the indicated time points. (**C**) A549 cells were pretreated with or without 10 μM of NAC or 2 μg/mL of OB-R antibody for 1 h and then stimulated with 1 μg/mL of leptin for another 1 h. The accumulation of intracellular ROS was detected by 2′,7′-Dichlorodihydrofluorescein diacetate (H2DCFDA) assay. Data are shown as the mean ± SEM of five independent experiments. ^&^ *p* < 0.05 as compared with the group of 0 min or basal group. * *p* < 0.05 as compared between the two indicated bars.

ROS play a link between physiology and pathology. Unbalanced ROS contribute to inflammatory gene expression [30]. To evaluate whether ROS are involved in leptin-mediated cPLA2α and COX-2 expression, mice were intraperitoneally (i.p.) injected with the ROS scavenger *N*-acetylcysteine (NAC), and leptin was then applied via intra-tracheal (i.t.) injection. After the mice were sacrificed, lung slices were prepared for IHC staining. As shown in Figure 2A, the pretreatment of NAC decreased leptin-mediated cPLA2α and COX-2 protein expression in lung tissue. Alveolar type II cells were used as a cell model to test the effect of leptin. Cells were incubated with leptin for the indicated time points and intracellular ROS were detected by 2′,7′-Dichlorodihydrofluorescein diacetate (H2DCFDA) assay. Leptin stimulated intracellular ROS accumulation in a time-dependent manner (Figure 2B). Pretreatment of NAC or OB-R antibody significantly reduced the leptin-mediated intracellular ROS increase (Figure 2C). These data suggest that leptin enhanced cPLA2α and COX-2 expression via OB-R-regulated ROS accumulation.

### 2.3. Activation of NADPH Oxidase Contributes to Leptin-Regulated cPLA2α and COX-2 Expression

**Figure 3:**
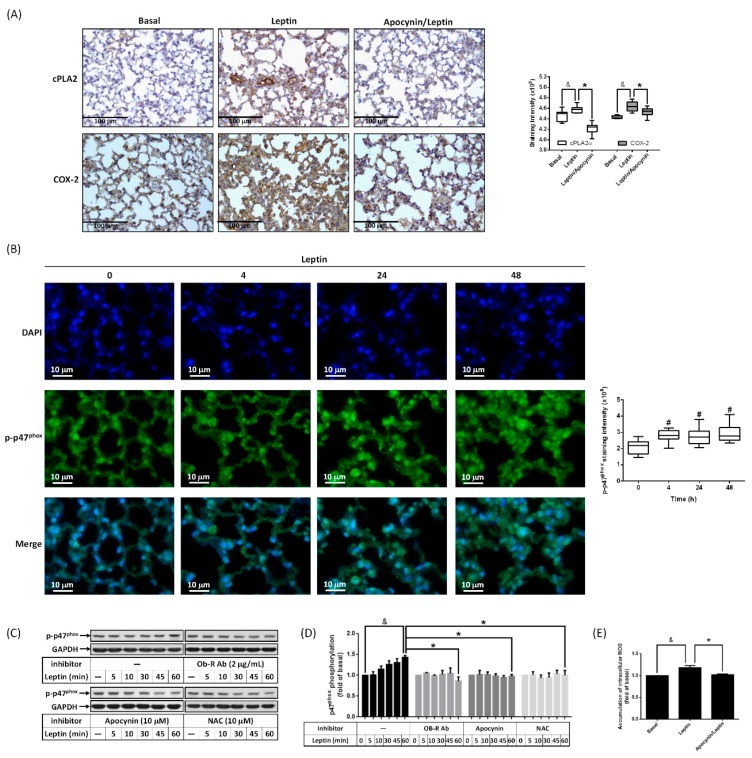
Leptin stimulated expression of cPLA2 and COX-2 via activation of NADPH oxidase. (**A**) ICR mice were i.p. injected with 2 mg/Kg of apocynin for 1 h then i.t. injected with 2 mg/Kg of leptin for 48 h. Expression of cPLA2 (top panel) and COX-2 (bottom panel) was shown by IHC. The staining intensities of cPLA2 and COX-2 are shown as box plots summarizing the median, 25th and 75th percentiles, whiskers, and outliers. (**B**) ICR mice were i.t. injected with 2 mg/Kg of leptin for 0, 4, 24, or 48 h. Phosphorylation of p47^phox^ (a subunit of NADPH oxidase) was detected by immunofluorescence staining. DAPI was used to localize the site of the nucleus. The staining intensity of p-p47^phox^ is shown as box plots summarizing the median, 25th and 75th percentiles, whiskers, and outliers. (**C**,**D**) Alveolar type II cells were pretreated with or without OB-R antibody (2 μg/mL), apocynin (10 μM), or NAC (10 μM) for 1 h and then stimulated with leptin (1 μg/mL) for the indicated time intervals. Phosphorylation of p47^phox^ was detected by Western blot. (**D**) Expression of p-p47^phox^ was quantified and is shown as a bar graph. (**E**) Cells were pretreated with or without apocynin (10 μM) and then incubated with leptin for 1 h. The accumulation of intracellular ROS was detected by H2DCFDA assay. Data are shown as the mean ± SEM of five independent experiments. ^#^ *p* < 0.05 as compared with the group of 0 min. ^&^ *p* < 0.05 or * *p* < 0.05 as compared between the two indicated groups.

Activation of NADPH oxidase promotes the generation of ROS and is involved in lung inflammation [24]. To elucidate whether NADPH oxidase correlated to the leptin-promoted expression of cPLA2α and COX-2, mice were i.p. injected with apocynin, an inhibitor of NADPH oxidase, followed by i.t. application with leptin. IHC staining revealed that leptin-regulated expression of cPLA2α and COX-2 was reduced by apocynin (Figure 3A), which suggested that the inhibition of NADPH oxidase attenuated cPLA2α and COX-2 expression in leptin-stimulated lungs. Data from immunofluorescence staining showed that stimulation with leptin up-regulated phosphorylation of p47^phox^, a subunit of NADPH oxidase, in the lung (Figure 3B). To confirm the effect of leptin on p47^phox^, cells were treated with leptin for the indicated time points, and Western blot was used to evaluate p47^phox^ phosphorylation in cell extracts. We found that leptin stimulated an increase in the p47^phox^ phosphorylation level in a time-dependent manner (Figure 3C,D). Blockage of OB-R, scavenging of ROS, or inhibition of NADPH oxidase significantly attenuated leptin-regulated p47^phox^ phosphorylation (Figure 3C,D). Moreover, ROS accumulation was also reduced by apocynin in leptin-treated cells, suggesting that leptin enhanced NADPH oxidase activation and produced ROS (Figure 3E). Briefly, these data imply that leptin promoted cPLA2α and COX-2 expression at least via OB-R-regulated NADPH oxidase activation and ROS production.

### 2.4. Involvement of AP-1 in Leptin-Mediated cPLA2α and COX-2 Expression

**Figure 4:**
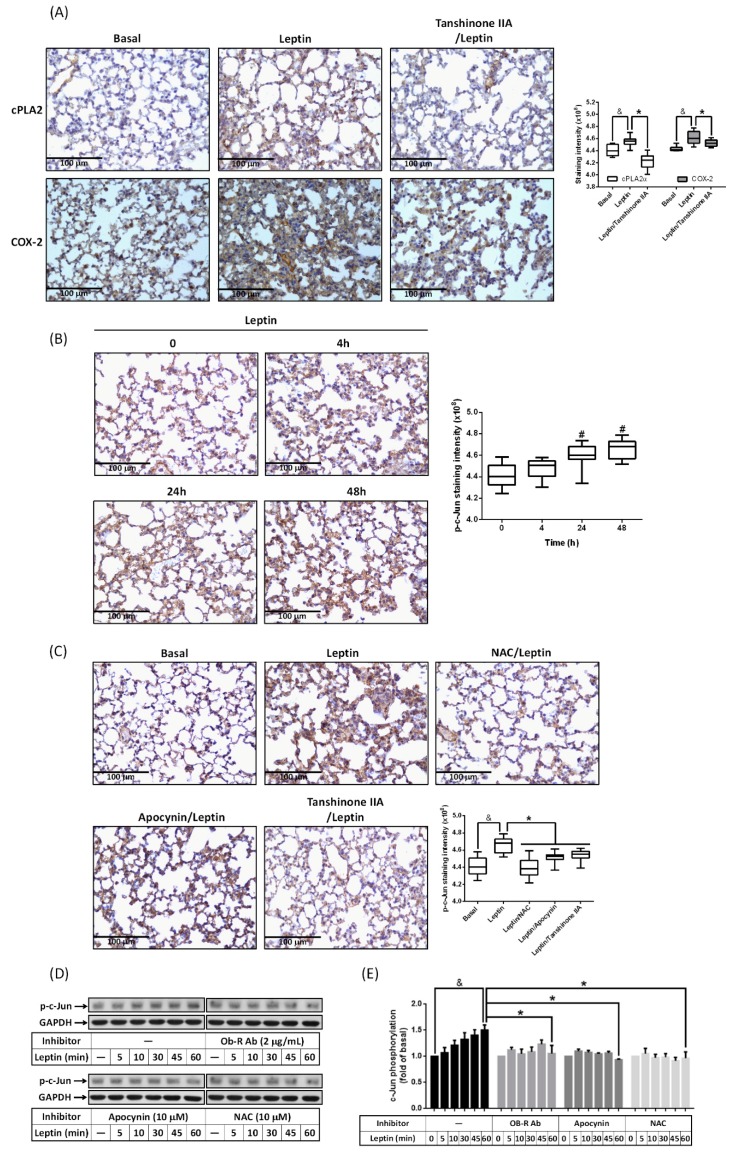
Involvement of AP-1 in leptin-stimulated cPLA_2_ and COX-2 expression. ICR mice were i.t. injected with 2 mg/Kg of leptin for the indicated time intervals. Alternatively, mice were i.p. injected with 2 mg/Kg of tanshinone IIA for 1 h then i.t. injected with 2 mg/Kg of leptin for 48 h. (**A**) Expression of cPLA2 (**top**) or COX-2 (**bottom**) and (**B**,**C**) phosphorylation of c-Jun were detected by IHC. The staining intensity of IHC data is shown as box plots summarizing the median, 25th and 75th percentiles, whiskers, and outliers. A549 cells were pretreated with or without the OB-R antibody (2 μg/mL), NAC (10 μM), or apocynin (10 μM) for 1 h and then stimulated with leptin (1 μg/mL) for the indicated time intervals. (**D**) Phosphorylation of c-Jun (a subunit of AP-1) was detected by Western blot. (**E**) Phosphorylation levels of c-Jun are shown as the mean ± SEM of five independent experiments. ^#^ *p* < 0.05 as compared with the group of 0 min. ^&^ *p* < 0.05 or * *p* < 0.05 as compared between the two indicated groups.

Several studies have indicated that AP-1 is one of transcriptional regulators in cPLA2α and COX-2 genes [8,25]. To study whether activation of AP-1 participated in leptin-regulated cPLA2α and COX-2 expression, tanshinone IIA, an inhibitor of AP-1, was i.p. injected into mice. IHC staining showed that the expression of cPLA2α and COX-2 was reduced in the tanshinone-IIA-treated group compared with the group receiving leptin alone (Figure 4A). It also showed that leptin increased the phosphorylation of c-Jun, a subunit of the AP-1 complex, in a time-dependent manner (Figure 4B). Leptin-enhanced c-Jun phosphorylation was attenuated by tanshinone IIA (Figure 4C). Moreover, pretreatment with NAC or apocynin also down-regulated the phosphorylation of c-Jun in leptin-treated lung (Figure 4C). These observations suggest that leptin may modulate AP-1 activity via NADPH oxidase-dependent ROS production. Indeed, in the cell model, leptin increased the phosphorylation of c-Jun in a time-dependent manner (Figure 4D,E). Pretreatment with an OB-R blockage antibody, apocynin, or NAC significantly reduced p-c-Jun expression in leptin-stimulated cells (Figure 4D,E). Collectively, these data reveal that leptin promoted cPLA2α and COX-2 expression via OB-R/NADPH oxidase/ROS-regulated phosphorylation of c-Jun.

### 2.5. Leptin Enhances Leukocyte Recruitment in Lung Space

**Figure 5:**
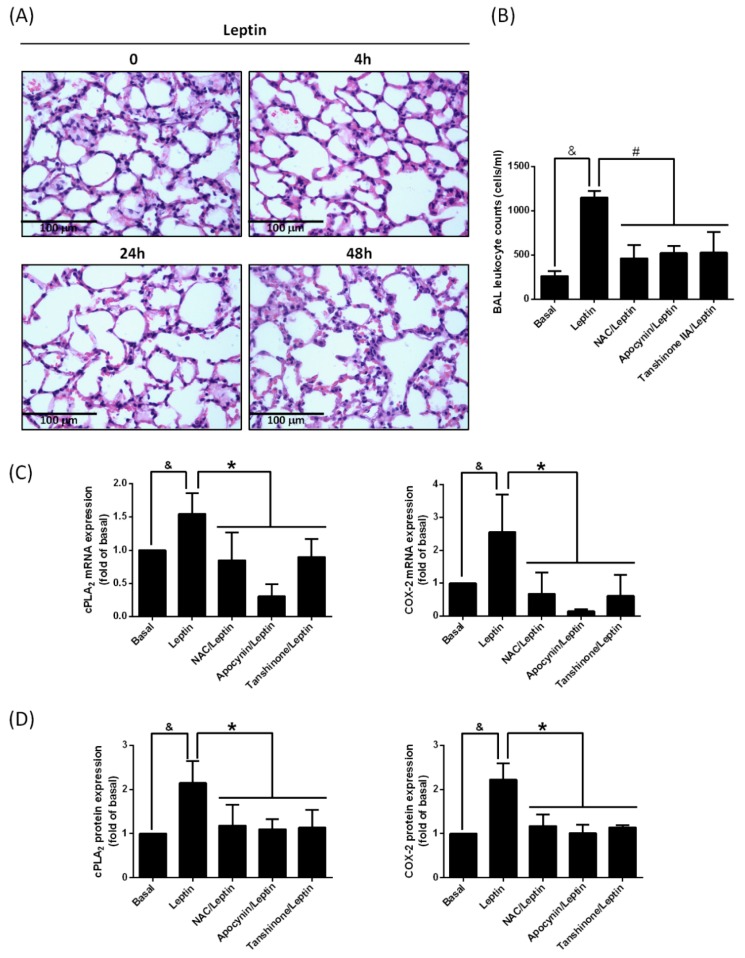
Leptin modulated accumulation of leukocytes and increased expression of cPLA2 and COX-2 genes in lung tissues. (**A**) Mice were i.t. injected with leptin (2 mg/Kg) for the indicated time intervals. Paraffinized lung tissues were sectioned and H&E staining was performed. (**B**) ICR mice were i.p. injected with 2 mg/Kg of NAC, apocynin, or tanshinone IIA for 1 h. Then, mice were i.t. injected with 2 mg/Kg of leptin for 48 h. Bronchoalveolar lavage (BAL) were saved and numbers of leukocytes were calculated using a scil Vet ABC™ Hematology Analyzer. (**C**) mRNA were extracted from lung tissues. RT-PCR was used to detect the mRNA expression of cPLA2 and COX-2. (**D**) Protein were extracted from lung tissues and Western blot was used to detect the expression of cPLA2 and COX-2. Data are expressed as the mean ± SEM of five independent experiments (*n* = 5). ^&^ *p* < 0.05 or * *p* < 0.05 as compared between the two indicated groups.

Leukocyte recruitment is a characteristic of tissue inflammation [12]. To discriminate whether the stimulation of leptin resulted in lung inflammation, lung tissue slices were stained after leptin application. As shown in Figure 5A, increased leptin in the lung resulted in increased blood cells in the alveolar space, showed by the haemotoxylin and eosin (H&E) stain (Figure 5A). The collection of Isolation of bronchoalveolar lavage (BAL) was used to quantify the amounts of leukocytes. As shown in Figure 5B, the stimulation with leptin increased the recruitment of leukocytes in the bronchoalveolar space. Treatment with NAC, apocynin, or tanshinone IIA significantly reduced the leukocyte recruitment (Figure 5B), suggesting that blockage of the signaling molecules underlying cPLA2α/COX-2 expression reversed leptin-regulated inflammation. Indeed, treatment with NAC, apocynin, or tanshinone IIA significantly decreased cPLA2α and COX-2 mRNA, and protein expression in leptin-stimulated lungs (Figure 5C,D and Supplemental Data 1). This revealed that the abolishment of NADPH oxidase/ROS-dependent AP-1 activation reversed leptin-mediated cPLA2α and COX-2 gene expression in the lungs.

## 3. Discussion

Obesity predisposes patients to the exacerbation of acute and chronic lung diseases. The morbidity of ARDS/acute lung injury (ALI) is higher in obese patients than in those of normal weight [2]. Treatment with obese serum containing leptin was shown to lead to increased susceptibility to lipopolysaccharide-induced lung injury via increased adhesion molecule expression and down-regulated junctional protein expression [4]. Restoring serum lean adipokine (adiponectin) levels reversed the effects of obesity on the lung endothelium and attenuated the susceptibility to acute injury [4]. Similarly, moderate to severe COPD patients with obesity have a worse COPD-related course and increased morbidity [6]. Although the correlation of obesity with pulmonary diseases has been previously reported, the molecular mechanisms have not been fully studied. Herein, we showed that the in vivo application of leptin to the lung system increased the gene expression of cPLA2α and COX-2 and leukocyte recruitments in lung tissues. Pretreatment with an ROS scavenger, NADPH oxidase inhibitor, or AP-1 inhibitor significantly reduced cPLA2α/COX-2 expression and leukocyte accumulation in the lung. Moreover, leptin application increased the production of ROS in an NADPH oxidase-dependent manner and resulted in the activation of AP-1. In summation, leptin increased lung cPLA2α/COX-2 expression and leukocyte recruitment in an NADPH oxidase/ROS/AP-1 manner.

As reported, abdominal obesity-related metabolic syndrome is associated with greater lung function impairment [31]. Accumulation of fat in the mediastinum and thoracic cavities alters the mechanical properties of the lungs and chest [32]. In addition, the immune responses of the respiratory system change under the various effects of adipokines. Leptin is a dominant cytokine released by adipose tissues and is correlated to airway reactivity in obese subjects with asthma [33]. Previous studies have revealed that the leptin levels in the BAL of obese mice are significantly increased compared to in lean mice [34]. Application of the peroxisome proliferator activated receptor gamma (PPARγ) Ligand rosiglitazone further increased the leptin concentrations in BAL [34], suggesting that treatment of obesity-related type II diabetes may result in higher leptin levels in the lung system. Herein, we report that increased levels of leptin in the lung result in the upregulation of the inflammatory proteins cPLA2α and COX-2 and promote the accumulation of leukocytes. It is suggested that the expression of cPLA2α or COX-2-dependent prostaglandin E2 (PGE2) exacerbates Streptococcus pneumoniae or Escherichia coli-infected damage to the lung and endometrium [35,36]. Moreover, persistent chronic inflammation leads to fibrosis [37,38]. In fact, leptin is highly deposited in the lung tissues of idiopathic pulmonary fibrosis and is positively correlated with the high-resolution computed tomography (HRCT) scores of patients [39].

The accumulation of ROS contributes to the occurrence or amplification of inflammation. ROS have been found to facilitate the virus replication and infection of influenza in lung epithelial cells [40]. Fine particulate matter significantly up-regulate pro-inflammatory mediators, including tumor necrosis factor (TNF)-α and IL-1β expression, via increased oxidative stress in rat lungs [41]. It was found that the activation of NADPH oxidase in alveolar epithelial cells is a source of ROS production [42]. Inhibition of NADPH oxidase by apocynin or diphenyleneiodonium chloride reversed TNF-α-mediated cPLA2α expression [43]. Here, we found that the stimulation of leptin promoted p47^phox^ phosphorylation and ROS production in an OB-R-dependent manner. Treatment with apocynin or NAC attenuated leptin-induced cPLA2α/COX-2 expression together with leukocyte recruitment, indicating that leptin mediated lung inflammation via OB-R-dependent NADPH oxidase-derived ROS. These results resemble those found for A549 cells in which carbon black nanoparticles enhanced oxidative stress by upregulation of NADPH oxidase (Nox) 2 and membrane expression of p67^phox^ accompanied with an increase of ROS production [44]. The absence of NADPH oxidase in mice increases H3N2 influenza virus clearance, reduces lung damage, and improves lung function [45].

c-Jun, belonging to the AP-1 family, is a member of the early mammalian transcriptional regulators and is involved in the regulation of tissue inflammation [46]. It has been reported that AP-1 is activated in response to increased intracellular oxidative stress and is critical to the transcription of proinflammatory genes such as IL-8, IL-6, and TNF-α [47]. Here, leptin was shown to increase the phosphorylation of c-Jun both in vivo in ICR mice and in vitro in A549 cells, and treatment with an AP-1 inhibitor reduced leptin-regulated cPLA2α and COX-2 expression. Moreover, pretreatment with OB-R, NAC, or apocynin attenuated leptin-mediated c-Jun phosphorylation, suggesting that leptin stimulated cPLA2α and COX-2 expression via OB-R/NADPH oxidase/ROS-activated c-Jun. In fact, AP-1 is involved in β-glucan-mediated IL-1β secretion in B-lymphocytes, suggesting important roles in the exacerbation of inflammation [48]. Alleviation of AP-1’s effects may contribute to the reduction of pulmonary injury [49].

**Figure 6:**
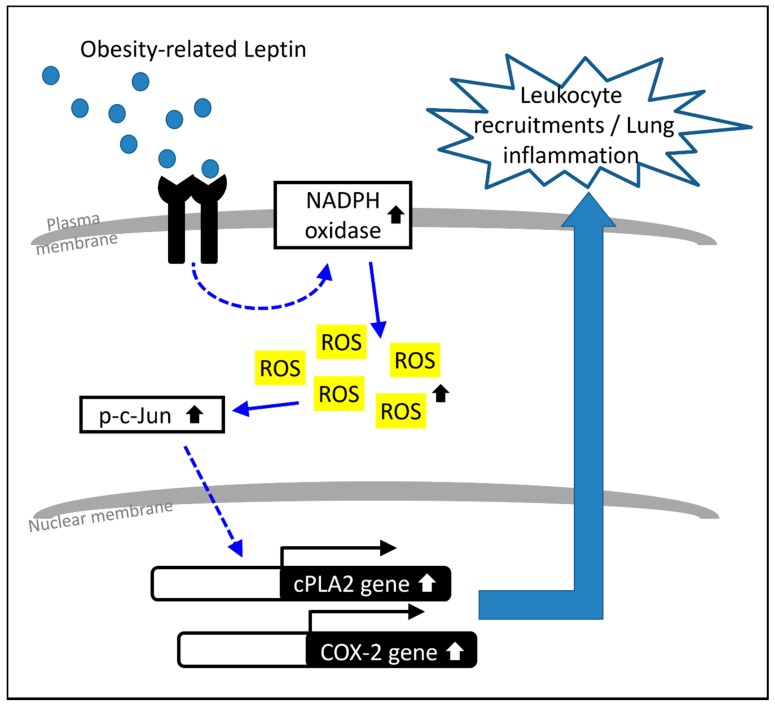
Leptin increased the expression of cPLA2/COX-2 together with accumulation of leukocytes via the NADPH oxidase/ROS/AP-1 pathway. According to the results and literature surveys, leptin promoted the activation of NADPH oxidase, resulting in the accumulation of intracellular ROS. The increase of oxidative stress contributed to the phosphorylation of c-Jun in leptin-stimulated lung tissue. In response to the leptin stimulation, activated AP-1 upregulated the expression of cPLA2 and COX-2 genes, contributing to leukocyte recruitment and lung inflammation. The solid arrows represented a step in an activating pathway. Dashed arrows represented the uncertain mechanism that needs to be evaluated in the future.

Based on the literature and our findings, as depicted in Figure 6, our results demonstrate that increased leptin in the lung system induced cPLA2α/COX-2 expression and leukocyte infiltration via the NADPH oxidase-dependent production of ROS. The increase of OB-R-dependent intracellular oxidative stress promoted the phosphorylation of c-Jun, resulting in the transcription of cPLA2α and COX-2 genes. Blockage of the signaling conduction of OB-R/NADPH oxidase/ROS/AP-1 resulted in a reduction in cPLA2α/COX-2 expression and leukocyte accumulation in leptin-stimulated lung tissues. Altogether, the results of this study provide a molecular mechanism for the inflammatory effect of leptin in the lung and suggest that leptin functions as a link between obesity and lung inflammation.

## 4. Materials and Methods

### 4.1. Materials

DMEM/F-12 medium, fetal bovine serum (FBS), 5-(and-6)-chloromethyl-2′,7′-dichlorodihydrofluorescein diacetate, acetyl ester (CM-H2DCFDA), DAPI, and Trizol were purchased from Invitrogen (Carlsbad, CA, USA). Antibodies against cPLA2α, COX-2, phospho-c-Jun, GAPDH, and OB-R were purchased from Santa Cruz Biotechnology (Santa Cruz, CA, USA). Phospho-Plus p47^phox^ was acquired from Assay Biotechnology (Sunnyvale, CA, USA). *N*-acetylcysteine (NAC), apocynin (APO), and tanshinone IIA were purchased from Biomol (Plymouth Meeting, PA, USA). Leptin was acquired from BioVision (Milpitas, CA, USA). Hybond C membrane and Hyperfilms were acquired from GE Healthcare Biosciences (Buckinghamshire, UK). An enhanced chemiluminescence (ECL) Western blotting detection system was purchased from Visual Protein Biotechnology Co (Taipei, Taiwan). Enzymes and other chemicals were purchased from Sigma (St. Louis, MO, USA).

### 4.2. Animal Treatment

Male ICR mice aged 6 weeks were purchased from the BioLASCO Taiwan Co., Ltd. (Taipei, Taiwan) and handled according to the guidelines of the Animal Care Committee of Fu Jen Catholic University (IACUC number: A10257) and NIH Guides for the Care and Use of Laboratory Animals. ICR mice were anesthetized with pentobarbital (60 mg/Kg) i.p. and placed individually on a board in a near-vertical position, and the tongues were withdrawn with lined forceps. Leptin (2 mg/Kg) was placed posterior in the throat and aspirated into the lungs. Control mice were administrated sterile 0.1% BSA. Mice were unassisted after 10–20 min. After leptin treatments, mice were sacrificed and lung tissues were extracted to analyze protein and mRNA expression of cPLA2α, COX-2, GAPDH, or β-actin. Otherwise, the lung tissues were paraffin embedded for H&E staining, IHC staining, or immunofluorescence assay.

### 4.3. Isolation of Bronchoalveolar Lavage (BAL)

After leptin treatment for 4, 24, or 48 h, mice were anesthetized and BAL was performed through a tracheal cannula using 1 mL aliquots of ice-cold PBS medium. BAL samples were centrifuged at 500× *g* at 4 °C, and cell pellets were washed and re-suspended in PBS. Cell counts were determined using a scil Vet ABC™ Hematology Analyzer (scil animal care company Inc., IL, USA).

### 4.4. Cell Culture of Human Alveolar Epithelial Cell Carcinoma (A549)

A549 cells, human alveolar epithelial cell carcinoma, were a well-established drug metabolism model of type II pulmonary epithelial cells [50], and were cultured as previously described [8]. When the cultures reached confluence, cells were treated with 0.05% trypsin/0.53 mM EDTA for 5 min at 37 °C. The cell suspension was diluted with DMEM/F-12 containing 10% FBS to a concentration of 2 × 10^5^ cells/mL. The cell suspension was plated onto 12-well culture plates (1 mL per well) or 10 cm culture dishes (10 mL per dish) for the measurement of protein expression and mRNA accumulation.

### 4.5. Protein Extraction and Western Blot

After leptin stimulation, the cells were then rapidly washed with ice-cold PBS, scraped, and collected by centrifugation at 1000× *g* for 10 min. The collected cells were lysed with ice-cold lysis buffer. The lysates were centrifuged at 45,000× *g* for 1 h at 4 °C to yield the whole cell extract. Samples from these supernatant fractions (30 μg protein) were subjected to SDS-PAGE using a 10% or 12% running gel. Proteins were transferred to nitrocellulose membrane, and the membrane was incubated at room temperature with 5% BSA in Tween-Tris-buffered saline (TTBS) for 1 h. Membranes were incubated overnight at 4 °C with an anti-phospho-c-Jun, anti-phospho-p47^phox^, or anti-GAPDH antibody according to the recommendations of the manufacturer. Membranes were incubated with a 1:2000 dilution of anti-mouse or anti-rabbit horseradish peroxidase antibody for 1 h. The immunoreactive bands detected by ECL reagents were developed using Hyperfilm-ECL.

### 4.6. Total RNA Extraction and Gene Expression Analysis

Total RNA was extracted from lung tissues using Trizol as previously described [8]. The cDNA containing 2 μg RNA was used as templates to analyze the cPLA2α and COX-2 mRNA levels. The oligonucleotide primers for β-actin, cPLA2α, and COX-2 were as follows: for β-actin, 5′-TGACGGGGTCACCCACACTGTGCCCATCTA-3′ (Sense) and 5′-CTAGAAGCATTTGCGGTGGA-CGATG-3′ (Anti-sense); for cPLA2α, 5′-CTCACACCACAGAA AGTTAAAAGAT-3′ (Sense) and 5′-GCTACCACAGGCACATCA-CG-3′ (Anti-sense); for COX-2, 5′-TTCAAATGAGATTGTGGGAA-AATTGCT-3′ (Sense) and 5′-AGATCATCTCTGCCTGAGTATCT-3′ (Anti-sense). The amplification profile included one cycle of initial denaturation at 94 °C for 5 min; 30 cycles of denaturation at 94 °C for 1 min; primer annealing at 58 °C (cPLA2α), 62 °C (COX-2), and 60 °C (β-actin) for 1 min; extension at 72 °C for 1 min; and then one cycle of final extension at 72 °C for 5 min. The expression of β-actin was used as an internal control for the assay of a constitutively expressed gene.

### 4.7. ROS Measurement by CM-H2DCFDA Fluorescence

Formation of ROS in A549 cells was determined as previously described [51]. A549 cells (about 90% confluence in 10 cm dishes) were loaded with 1 mM DCF-DA for 30 min in PBS at 37 °C in a 95% air/5% CO2 environment. The medium containing DCF-DA was aspirated, and the cells were washed twice with PBS and replenished with 0.5 mL of DMEM/F-12 medium. Cells were pretreated without or with the inhibitors for 1 h followed by exposure to adiponectin for the indicated time intervals. The cells were washed twice with ice-cold PBS and scraped using a lysis buffer (1× PBS containing 20% alcohol and 0.1% Tween 20). The cell lysates were transferred to 1.5 mL Eppendorf vials and centrifuged at 10,000× *g* for 1 min at 4 °C. Fluorescence of oxidized DCF-DA in cell lysates, an index of formation of ROS, was measured at room temperature using an Infinite 200 PRO multimode reader (Tecan Group, Männedorf, Switzerland) with excitation and emission set at 490 and 530 nm, respectively.

### 4.8. Immunohistochemical Staining and Immunofluorescence Assay

Paraffinized lung tissues were deparaffinized, rehydrated, and washed with TTBS. Non-specific binding was blocked by pre-incubation with blocking solution for 1 h at room temperature. The sections were incubated with an anti-cPLA2α (1:200), anti-COX-2 (1:200), or anti-p-p47^phox^ (1:100) antibody at 4 °C for 16 h and then with anti-mouse HRP (horseradish peroxidase) or anti-rabbit-FITC antibody at room temperature for 1 h. Bound antibodies were detected by incubation in 0.5 mg/mL of 3,3-diaminobenzidine (DAB)/0.01% hydrogen peroxide in 0.1 M Tris-HCl buffer as the chromogen (Vector Lab, Burlingame, CA, USA).

### 4.9. Statistical Analysis of Data

All signaling intensities of IHC or immunofluorescence data are shown as box plots summarizing the median, 25th and 75th percentiles, whiskers, and outliers. Other data are shown as the mean ± the standard error of the mean using the GraphPad Prism Program (GraphPad, San Diego, CA, USA). Quantitative data were analyzed using a one-way ANOVA followed with Tukey’s post hoc test at a *p* < 0.05 level of significance. All of the experiments were performed at least five times.

## Supplementary Materials

Supplementary materials can be found at https://www.mdpi.com/1422-0067/20/5/1078/s1.
