# Supplementary figures and images for: Participation of NADPH Oxidase-Related Reactive Oxygen Species in Leptin-Promoted Pulmonary Inflammation: Regulation of cPLA2α and COX-2 Expression
Source: Int J Mol Sci. 2019 Mar 2;20(5):1078. doi: 10.3390/ijms20051078 (PMC6429300; doi:10.3390/ijms20051078)

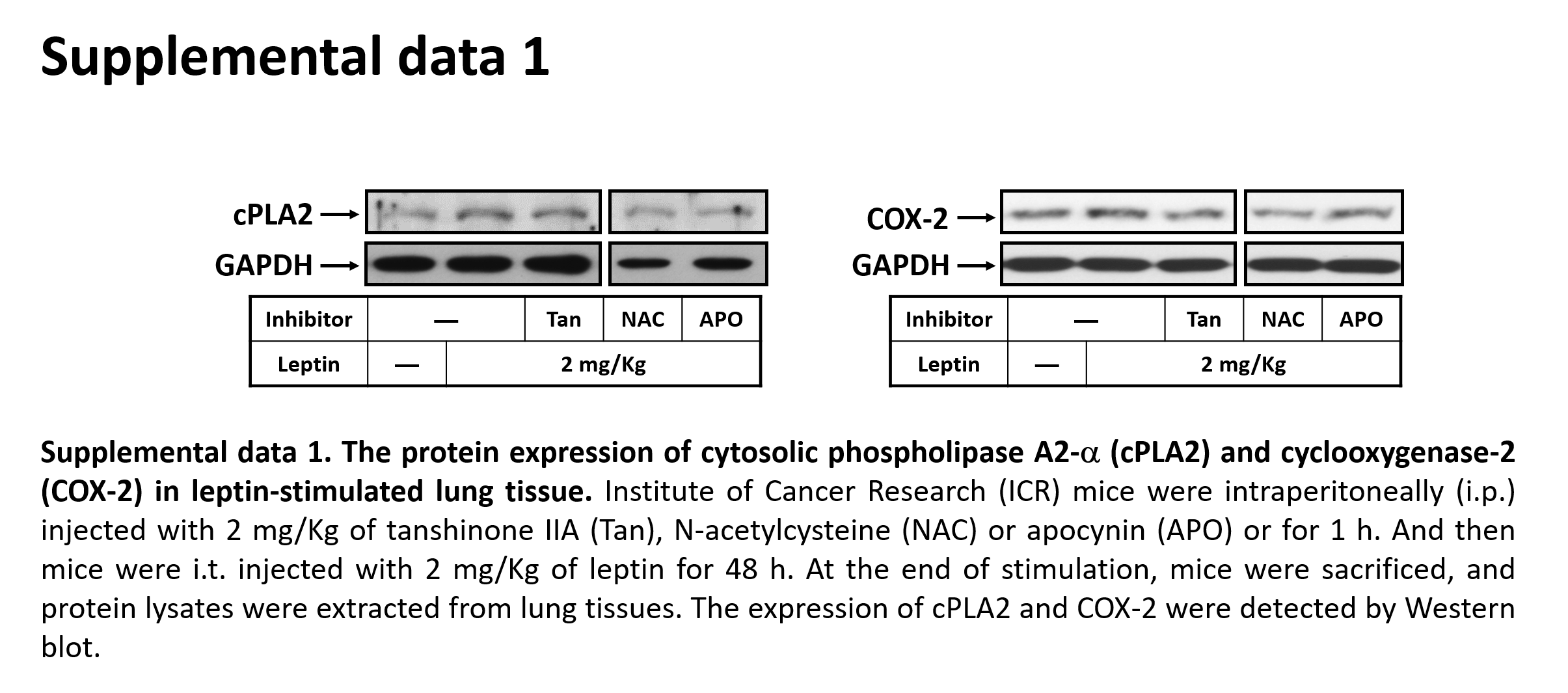

Supplement: Supplementary file 1 [file ijms-20-01078-s001.zip › ijms-441869-supplementary.tif]
